# Seasonal Influenza Vaccination in Health Care Workers. A Pre-Post Intervention Study in an Italian Paediatric Hospital

**DOI:** 10.3390/ijerph15050841

**Published:** 2018-04-24

**Authors:** Francesco Gilardi, Guido Castelli Gattinara, Maria Rosaria Vinci, Marta Ciofi Degli Atti, Veronica Santilli, Rita Brugaletta, Annapaola Santoro, Rosina Montanaro, Luisa Lavorato, Massimiliano Raponi, Salvatore Zaffina

**Affiliations:** 1 Occupational Medicine Unit, Bambino Gesù Children’s Hospital, IRCCS, 00146 Rome, Italy; francesco.gilardi@opbg.net (F.G.); mariarosaria.vinci@opbg.net (M.R.V.); rita.brugaletta@opbg.net (R.B.); annapaola.santoro@opbg.net (A.S.); luisa.lavorato@opbg.net (L.L.); 2 Vaccination Unit, University Hospital Paediatric Department, Bambino Gesù Children’s Hospital, IRCCS, 00146 Rome, Italy; guido.castelli@opbg.net (G.C.G.); veronica.santilli@opbg.net (V.S.); 3 Health Directorate, Bambino Gesù Children’s Hospital, IRCCS, 00165 Rome, Italy; marta.ciofidegliatti@opbg.net (M.C.D.A.); massimiliano.raponi@opbg.net (M.R.); 4 University Hospital Paediatric Department, Bambino Gesù Children’s Hospital, IRCCS, 00165 Rome, Italy; rosina.montanaro@opbg.net

**Keywords:** occupational health, health care workers, seasonal influenza vaccination

## Abstract

Despite relevant recommendations and evidences on the efficacy of influenza vaccination in health care workers (HCWs), vaccination coverage rates in Europe and Italy currently do not exceed 25%. Aim of the study is to measure the variations in vaccination coverage rates in an Italian pediatric hospital after a promotion campaign performed in the period October–December 2017. The design is a pre-post intervention study. The intervention is based on a wide communication campaign and an expanded offer of easy vaccination on site. The study was carried out at Bambino Gesù Children’s hospital in Rome, Italy, on the whole population of HCWs. Univariate and multivariate statistical analyses were performed. Vaccination coverage rate increased in 2017/18 campaign compared with the 2016/17 one (+95 HCWs vaccinated; +4.4%). The highest increases were detected in males (+45.7%), youngest employees (+142.9%), mean age of employment (+175%), other HCWs (+209.1%), Emergency Area (+151.6%) and Imaging Diagnostic Department (+200.0%). At multivariate logistic regression, working in some departments and being nurses represents a higher risk of being unvaccinated. Although the vaccination coverage rate remained low, a continuous increase of the coverage rate and development of a different consciousness in HCWs was highlighted. The study significantly identified the target for future campaigns.

## 1. Introduction

Around the world seasonal influenza (SI) causes approximately 3–5 million severe cases and 500,000 deaths every year, through an increase of all-cause mortality [1,2]. The related high economic costs are also connected with workers’ absenteeism and an increased demand of health care services [3].

Health care workers (HCWs) are exposed to an increased risk to contract and diffuse infectious diseases to vulnerable patients and colleagues more frequently than the general population [4,5,6]. Many of these pathologies are vaccine-preventable diseases, including SI [7]. Specific recommendations and annual influenza vaccination campaigns were set in order to reach increasing coverage rate in HCWs.

Some studies estimate an annual incidence of infection in unvaccinated HCWs three times higher than in those vaccinated [8]. In many cases influenza in HCWs can be asymptomatic but transmissible to their patients [9].

The efficacy of seasonal influenza vaccination (SIV) has been demonstrated by a large number of evidences [10,11,12,13,14,15,16,17,18,19]. Its positive effects were mainly proved by the reduction of influenza transmission in healthcare settings, staff illness, absenteeism and influenza-related morbidity and mortality [10,11,20,21,22,23,24]. Its cost-effectiveness is also an added value [25].

HCWs aging represents a further indication for vaccination and its positive impact on health status and productivity. Moreover, HCWs are the main referents for the vaccine related information [26] and the first main promoters for their patients and colleagues in the challenge to the phenomenon of “vaccine hesitancy” [27,28,29,30].

In spite of these evidences and the continuous recommendations made over the last decades by the main health authorities [26,31,32,33,34], in European Countries the median of vaccination coverage rate (VCR) in HCWs decreased from 26% in the season 2007/2008 to 25.7% in the season 2014/2015 with a wide variation among countries (i.e., UK 53%, Poland 5%) [26]. As for Italy, several local studies recorded a VCR of about 20% [35,36,37,38,39,40]. These European data are largely insufficient if compared with those detected in the USA [32], where SIV is mandatory with a high coverage rate (HCWs over 70%, physicians over 90%, nurses 85%).

The alternative to the mandatory vaccination, which many stakeholders recommend [7,41,42,43] and seems to be the only feasible in Europe—is the adoption of new strategies and interventions [31] based on behaviour change, scientific evidence and integrated health program planning models [30,44].

At local level the implementation of the best practice and behaviour change oriented programs can be accompanied by the easy access to vaccination [45,46,47], as well as by education and promotion actions based on impact analysis of SI in HCWs in each health service (hospital, primary care, long term care, etc.). The aim of the study was to measure VCR variations of HCWs after a specifically dedicated campaign of communication carried out for SIV in the period October–December 2017 in a paediatric hospital.

## 2. Methods

### 2.1. Study Design

A pre-post intervention study was carried out comparing the annual VCR in the HCWs of the hospital between the last two SIV campaigns (2016/2017; 2017/2018).

### 2.2. Setting

The study was carried out at Bambino Gesù Children’s Hospital (OPBG), a large paediatric research hospital with more than 600 settled-bed, located in Rome, Italy, accredited since 2007 by the International Joint Commission. It is considered a reference hospital for paediatric diseases in central and southern Italy with a total of 27,342 ward admissions in the year 2014. More than one million and a half procedures were administered in the outpatient clinics in 2014. Finally, about 77,000 patients were cared in the Emergency and First Aid Department.

### 2.3. Data Collection, VCR and Variables Considered

Data about SIV were collected from the database of the hospital Vaccination Unit and Occupational Medicine regarding the last two years. Data about HCWs were retrieved from the database of the hospital Human Resources Direction and recorded by gender, age group, job category, length of employment, hospital area and department. Annual VCRs were evaluated at the hospital level, in 2016 and 2017, in order to verify the change of HCWs attitude toward SIV. Annual VCRs were estimated by using the number of SIV doses administered to HCWs as the numerator, and the total number of OPBG HCWs of permanent staff at the end of each year as the denominator.

### 2.4. The Intervention of SIV Promotion. Communication Campaign and Vaccination Offer

Over the course of the IV quarter of 2017 (October–December 2017), a wide and multilevel intervention of vaccination campaign was performed. Planned on communication, health education and promotion, as well as an easy access to vaccination, the campaign was carried out on the whole hospital personnel, promoted by the hospital management in collaboration with the Vaccination Unit and Occupational Medicine. Based on previous experiences started in 2009, free influenza vaccination was offered on-site to HCWs in the various units and departments through mobile teams [48].

The main new components of the SIV 2017/2018 campaign were:

- A wider offer of on-site vaccination
- A new vaccination offer during the surveillance occupational medicine visits
- A daily availability and prolonged time of vaccinations for HCWs in fixed stations at each hospital site
- An expanded and integrated communication strategy
- Social promotion initiatives.

In comparison with the 2016/2017 campaign, special attention and promotion was posed in the more critical wards and for OHCWs who showed lower acceptance rates. The on-site vaccination was offered to a wider number of Units and Departments and with the constant presence of an occupational physician. Moreover, dedicated vaccination sessions during the surveillance visits of the Occupational Medicine were offered. The campaign has provided a fixed vaccination station for the HCWs, for each of the four hospital sites, every day, with a dedicated time slot.

The campaign was organized through several media and tools. Information and promotion materials, such as advertising posters, letters and personal solicitations were sent and directed to all hospital wards, all medical and department directors and posted on the hospital’s intranet. The communication tools and their diffusion consisted in: (a) posters, placed in areas which were frequently visited by HCWs (changing rooms, badge area, canteen, etc.); (b) information factsheets, distributed in paper form in hospital wards and also available in electronic format on the hospital’s intranet; (c) a banner on the hospital’s intranet, linked to the electronic information factsheets.

The campaign key messages have been focused on patient and personal protection (i.e., “Protect your patients! Protect yourself!”, “Be prepared. Get vaccinated!”, “Get informed! Get vaccinated! Get protected!”). Vaccinated HCWs were asked to wear a pin, as testimony, with the inscription “I am vaccinated to protect children. And you?”. Moreover, education sessions for HCWs on the epidemiological data and critical aspects relative to the lack of influenza vaccination for vulnerable and at risk patients and the efficacy and effectiveness of influenza vaccination have been carried out during the campaign period.

Social promotion initiatives were integrated in the strategy of the campaign. During the SIV 2017/2018 campaign, in fact a special and informal competition was launched to identify the department which would have reached the best VCR. The competition was advertised on the home page of the hospital website of the hospital website through messages and interviews (Directors, Occupational Medicine and Vaccinations Service). Moreover, the initiative “Take a selfie while you get vaccinated” has helped promote the dissemination of good examples and prompt other HCWs to vaccination. On the basis of the percentage of vaccinated HCWs at 31 December 2017, the best Department was awarded with a commemorative plaque on the occasion of the hospital Quality Day (an event dedicated to sharing good practices to promote quality and safety of care).

### 2.5. Statistical Analysis

Univariate and multivariate analyses, as well as Student’s *t*-test for continuous variables and Chi-square test for categorical ones, were performed. SPSS Statistics software (IBM SPSS Statistics V20, Chicago, IL, USA) was used. Approval of the Ethics Committee was not required for this study as there was no intervention on participants and written informed consent was obtained by all HCWs.

## 3. Results

The population in study was composed by 2123 HCWs (2016/2017 campaign) and 2131 HCWs (2017/2018 campaign) in stable employment at the end of the IV quarter of 2016 and 2017. Little differences, due to missing records, have been highlighted between the samples. Females were 1579 in 2016 (74.4%) and 1,581 in 2017 (74.2%), males 544 in 2016 (25.6%) and 560 in 2017 (25.8%). The mean age of HCWs was 46.9 (SD ± 10.33) in (2016/17 campaign) 47.7 (SD ± 10.23) in 2017/18 one. 

At hospital level the number of doses of SIV administered to HCWs increased from 274 (12.9%) in the 2016/17 SIV campaign to 369 (17.3%) in the 2017/18 one. The total increase in 2017 was of 95 vaccinated HCWs (+34.1% with respect to the 2016/2017 vaccination rate). The vaccinated HCWs for the first time (at least not those vaccinated in the previous 2016/17 campaign) were 199 (53.9%) in 2017/2018 campaign. Vaccinated HCWs were 170 (46.1%) in both campaigns. HCWs who got vaccinated only in the 2016/2017 campaign were 104 (38%). VCR in new HCWs employed over the course of 2017 (not considered in the present study) was higher (23/108; 21.3%) than that recorded in those HCWs included in the study.

The mean age in vaccinated HCWs was 50.2 (SD ± 9.92) in 2016/17 and 49.4 (SD ± 10.36) in 2017/18 (*p* = 0.3). The mean age in unvaccinated HCWs was 46.4 (SD ± 10.30) in 2016/17 and 47.3 (SD ± 10.17) in 2017/2018 (*p* < 0.001).

The highest variation in age groups has been observed in the 20–30 age group (+142.9%), the lowest in the 51–60 one (+8.3%).

The highest variation in gender was recorded in males (+45.7%) (Table 1). Males are approximately a quarter of all HCWs in the hospital (25.7%).

The mean length of employment of hospital HCWs was 17.7 (SD ± 12.3). The mean length of employment in vaccinated HCWs and unvaccinated HCWs was the same 18.2 (SD ± 12.1) in the 2017/2018 campaign and 19.5 (SD ± 12.1) and 17.7 (SD ± 12.1) respectively in the 2016/2017 one. The highest variation for length of employment is within 6–10 year employees (+63.9%) and the lowest in over 20 year employees (+15%).

The Medical Paediatric Area (general paediatric and paediatric specialty units) reached the highest VCR in both the SIV campaigns (25.7% in 2016/17; 26.3% in 2017/18). The lowest VCR was recorded in Emergency Area (emergency and intensive care units) in 2016/17 (6.2%) and in Medical-Surgical Area (neurology/neurosurgery, neonatology/neonatal surgery, cardiology/cardiosurgery) in 2017/18 (11.8%). The highest increase was recorded in Emergency Department (+151.6%) and the lowest in Medical Paediatric Area (+2.3%) (Table 1).

The number of SIV doses increased in all job categories with a different distribution between physicians, nurses and OHCWs. The highest number of SIV doses were recorded in physicians both in 2016 (147; 28.4%) and in 2017 (162; 30.7%). The lowest result in SIV 2017/18 was recorded in nurses (10.7%). The job category which showed the highest increase was OHCWs (+209.1%). The lowest increase was reported in physicians (+8.1%) (Table 1).

The VCR increase in almost the Departments with variable differences in 2017 matched with 2016. In SIV 2016/17 and 2017/18 the highest VCR was recorded in University Hospital Paediatrics Department (31.2%; 29.9%). In 2016/17 SIV the lowest VCR was registered in Imaging Diagnostic Department (5.1%) and in 2017/18 SIV in Medical Surgical Cardiology (10.8%). The highest increase was recorded in Imaging Diagnostic Department (+200.0%) and the lowest in Health Directorate (−4.3%) (Table 1).

For all the variables considered (age group, gender, length of employment, job category, hospital area and hospital department) statistically significant differences in the distribution of VCR were observed both in 2016/2017 and 2017/2018 campaign (Table 1). Univariate analysis showed, comparing the VCR of the two years, statistical significant increases in several subgroups of all variables (Table 2).

At the multivariate logistic regression the variables statistically significant for the risk of being unvaccinated are: belonging to some Departments (Surgery, Diagnostic Imaging, Emergency, Medical Surgical Cardiology, Neonatology and Neuroscience and Rehabilitation), being nurses (OR 1.43, IC 95% 1.08–1.88) and having a length of employment between 6 and 10 years (OR 1.68, IC 95% 1.16–2.41) (Table 3). Being physicians is a factor predisposing to be vaccinated.

## 4. Discussion

After the SIV 2017/2018 campaign, VCR increased more than in campaigns carried out in the previous years: in fact, the improvement of vaccinated HCWs was +4.4%. This variation was more evident in specific groups, hospital areas and departments, such as males, youngest employees, OHCWs, Emergency Area and Imaging Diagnostic Department. Such a result shows that VCR in HCWs is increasing year by year following a progressive, although slow, gradual growing consciousness of HCWs toward influenza vaccination. Moreover, the high dedication of promotion campaign targeted to the most critical areas and lower responding HCWs seems to be the right methodology.

An important finding about the SIV 2017/2018 campaign was related to the higher number of vaccinated HCWs for first time (199/369 HCWs; 53.9%) compared to the old vaccinated ones. Due attention will be paid in order to understand the reasons connected with the high level of HCWs who did not undergo the new vaccination in the 2017/2018 campaign (104 HCWs 37.9% of those vaccinated in 2016/2017). Such evidence suggests that future campaigns should foresee recalls of vaccinated HCWs.

The campaign, promoted also by social initiatives, has proved to be more successful in new—and of course younger—employees who accepted to be vaccinated in a higher number than other colleagues (21.3%).

The components of the campaign that showed to be more effective and sustainable on HCWs vaccination has probably been the presence of the occupational physicians during the vaccination sessions. The possibility to receive adequate information, personal advices and counseling seems to have an important role in HCWs adhesion to vaccination, as proved in other public health practices.

This integrated program based on a wide communication plan, physician personal involvement and an annual expanded program of vaccination on-site vaccination facilities is proving its efficacy not only for HCWs but also for administrative and non-clinical or permanent staff (data not shown).

The best VCR, although not increased over the last year, was recorded in the medical area. It seems that a higher VCR, even if far below the target level, negatively affects the annual increase of vaccination acceptance. Pediatricians seem to have a different attitude toward vaccination and a better knowledge compared with colleagues of other specialties.

The results of the study are similar to several other surveys conducted in some Italian hospitals and health services [35,36,37,38,39,40]. The strength of the study is connected with the setting in which it was carried out: a large, reference and only paediatric hospital with all the different pediatric subspecialties. To the best of our knowledge, only a few studies on the issue have been reported so far in literature [49,50,51].

Further analysis will be necessary to evaluate the impact of influenza vaccination on HCWs’ absenteeism during the winter period.

## 5. Conclusions

Although such a VCR in a paediatric hospital is unsatisfying, the adoption of an extensive and agreed strategy at the different levels of hospital management showed its potential validity in changing HCWs attitude towards vaccinations. The analysis of VCR in the different hospital areas, job categories and departments allows to define some clear indications about the crucial aspects on which to focus the attention and the communication strategy of SIV campaign in the future. The present evaluation of our experience will allow to plan and integrate further tools, especially directed to those Departments and HCWs where the need of a higher VCR to protect fragile patients is of crucial importance. The integrated work involving the hospital Management, Vaccination Unit and Occupational Medicine in a synergic and coordinated manner has proven to be a good practice. The experience carried out over the last years about SIV and the demonstration of the effectiveness of coordinated and targeted actions is a valuable step for other missions in order to promote health actions and empowering the hospital personnel.

## 6. Limitations of the Study

The study analyzes only the last two SIV campaigns due to a new and higher availability of prompt updated data on vaccinations and HCWs. A comparison on the same information for the previous years could not be so precise. Moreover, another limitation of the study is due to the impossibility to evaluate all the unstructured personnel who work in the departments (volunteers, interpreters, cleaning staff, ancillary workers, students, attending physicians, etc.). However, this staff was also involved in the vaccination campaign and showed an increase of VCR (data not shown) similar to that detected in HCWs.

## Figures and Tables

**Table 1:** SIV coverage rate per age group, gender, hospital area, job category and hospital department.

Variables	2016/17				2017/18				Variations	
Age Group	SIV Doses	HCWs	%	*p*	SIV Doses	HCWs	%	*p*	Δ	Δ%
20–30	10	159	6.3	<0.001	17	111	15.3	<0.001	+7	+142.9
31–40	48	519	9.2		70	523	13.4		+24	+45.6
41–50	69	578	11.9		107	578	18.5		+39	+55.5
51–60	112	711	15.7		120	705	17.0		+8	+8.3
Over 60	35	156	22.4		55	214	25.7		+20	+14.7
**Gender**										
Female	179	1579	11.3	<0.001	229	1581	14.5	<0.001	+50	+28.3
Male	95	544	17.5		140	550	25.5		+45	+45.7
**Hospital Area**										
Medical PaediatricArea	98	381	25.7	<0.001	100	380	26.3	<0.001	+2	+2.3
Medical-Surgical Area	51	561	9.1		66	558	11.8		+15	+29.7
Emergency Area	21	341	6.2		47	302	15.6		+26	+151.6
Direction/Services Area	67	452	14.8		96	511	18.8		+29	+27.0
Surgical Area	24	251	9.6		41	270	15.2		+17	+58.3
Hematology Oncology	13	137	9.5		19	110	17.3		+6	+82.1
**Job category**										
Physicians	147	517	28.4	<0.001	162	527	30.7	<0.001	+15	+8.1
Nurses	102	1080	9.4		113	1053	10.7		+11	+13.8
OHCWs	29	526	5.5		94	551	17.0		+65	+209.1
**Departments**										
Medical Surgical Cardiology	18	221	8.1	<0.001	24	222	10.8	<0.001	+6	+33.3
Neuroscience and Rehabilitation	22	234	9.4		25	228	11.0		+3	+17.0
Imaging Diagnostic	6	118	5.1		19	124	15.3		+13	+200.0
Emergency/PICU	21	341	6.2		47	302	15.6		+26	+151.6
Medical Surgical Neonatology	11	106	10.4		17	108	15.7		+6	+51.0
Laboratories and Immunological Diagnostics	17	129	13.2		28	166	16.9		+11	+28
Surgery	24	251	9.6		41	270	15.2		+17	+58.3
Hematology Oncology	13	137	9.5		19	110	17.3		+6	+82.1
Paediatric University Hospital	64	206	31.2		65	217	29.9		+1	−4.2
Paediatric Medical Specialty	34	175	19.4		35	163	21.5		+1	+10.8
Health Directorate	36	155	23.		37	168	22.0		+1	−4.3
Scientific Direction	8	50	16.0		12	53	22.6		+4	41.3

**Table 2:** VCR variation.

Variables	% 2016/2017	% 2017/2018	Chi-Square (*p*)
Age group			
20–30	6.3	15.3	0.02
31–40	9.2	13.4	0.03
41–50	11.9	18.5	0.002
Gender			
Female	11.3	14.5	0.008
Male	17.5	25.5	0.001
Hospital Area			
Emergency Area	6.2	15.6	<0.001
Surgical Area	9.6	15.2	0.05
Job category			
Physicians	28.4	30.7	0.04
OHCWs	5.5	17.0	0.001
Departments			
Imaging Diagnostic	5.1	15.3	0.009
Emergency/PICU	6.2	15.6	<0.001
Surgery	9.6	15.2	0.05
Length of employment			
2–5 years	11.6	17.7	0.05
6–10 years	8.8	14.1	0.02
11–20 years	14.6	19.6	0.03

**Table 3:** Multivariate Logistic Regression.

Variables	*p*-Value	OR	95% CL	
			Lower	Upper
OHCWs	<0.001	1		
Physicians	<0.001	0.35	0.26	0.47
Nurses	0.01	1.43	1.08	1.88
Scientific Direction	<0.001	1		
Surgery Department	0.005	2.37	1.30	4.32
Imaging Department	0.002	2.88	1.48	5.62
Emergency Department	0.003	2.44	1.34	4.44
Med. Sur. Cardiology Department	0.002	2.75	1.47	5.15
Neuro Rehab Department	0.003	2.46	1.35	4.47
Over 20 years employed	<0.001	1		
6–10 years employed	<0.005	1.68	1.16	2.41
